# How Does Bereavement Affect the Health-Related Quality of Life of Household Members Who Do and Do Not Provide Unpaid Care? Difference-in-Differences Analyses Using the UK Household Longitudinal Survey

**DOI:** 10.1007/s40273-024-01452-1

**Published:** 2024-12-05

**Authors:** Becky Pennington, Mónica Hernández Alava, Mark Strong

**Affiliations:** Sheffield Centre for Health and Related Research, School of Medicine and Population Health, University of Sheffield, Sheffield, S1 4DA England

## Abstract

**Background:**

Guidelines for modelling in economic evaluation recommend that it may be necessary to consider costs and outcomes until all modelled patients have died. Some guidelines also recommend that carers’ health-related quality of life (HRQoL) outcomes should be included. However, it is unclear whether economic evaluations should continue to include carers’ HRQoL after patients have died, and whether there is any evidence to support an additional bereavement effect for carers.

**Methods:**

We used the UK Household Longitudinal Study waves 1–12. We used Difference-in-Differences to estimate the short- and long-term bereavement effects on the SF-6D for people who reported that they did and did not provide care to a household member who then died. We assumed parallel trends conditional on age, sex, long-term health conditions, education, and household income.

**Results:**

Carers and non-carers experienced a significant loss in HRQoL in the year immediately following bereavement. Carers potentially experienced a loss in HRQoL in the year before bereavement, whereas the bereavement effect may have lasted longer for non-carers. For both groups, HRQoL became comparable to the non-bereaved population around 3 years after bereavement.

**Conclusions:**

Bereavement has a statistically significant negative impact on HRQoL in the short-term, for both carers and non-carers. However, the effect size is small and is not sustained, suggesting that including bereavement in economic evaluation would make little difference to results.

**Supplementary Information:**

The online version contains supplementary material available at 10.1007/s40273-024-01452-1.

## Key Points for Decision Makers

**Table:** 

Bereavement is associated with a statistically significant health-related quality of life (HRQoL) loss in the first year—a disutility ranging from approximately 0.03 to 0.04 using SF-6D.
The size of HRQoL loss associated with bereavement is similar for carers and non-carers.
There is no sustained effect on HRQoL for either carers or non-carers beyond 2 years after bereavement.

## Introduction

Guidelines for modelling in health economic evaluation recommend that time horizons should be long enough to reflect all important differences between technologies [1], and that where technologies lead to differences in survival or lifelong benefits, it is necessary to consider costs and outcomes until all modelled patients have died [2, 3]. Guidelines are also increasingly recommending or permitting the inclusion of outcomes for people who provide unpaid care to patients, but recommendations on the appropriate time horizon for carers are less clear; for example, the Spillovers in Health Economics Evaluation and Research taskforce recommend that the time horizon should be sufficient to capture all relevant impacts including bereavement [4], but the Zorginstituut Nederland recommends that carers’ outcomes should only be included until the patient dies [5]. The Institute for Clinical and Economic Review in the US recognises the challenges in determining whether to include outcomes for carers after patients have died [6]. This is due to the potential for carers’ health-related quality of life (HRQoL) to improve after the person they care for dies, such that an intervention that extends patient survival can lead to a quality-adjusted life-year (QALY) gain to patients but a QALY loss to carers (termed the “carer QALY trap” by Mott et al. [7]). This situation arises because economic evaluations typically model carers as having lower HRQoL than people who do not provide care. After the patient dies, it is assumed that the (former) carer no longer experiences the detrimental effect of caregiving, and so their HRQoL becomes comparable to that of people who never provided care. While there has been much discussion as to the implications and interpretation of this assumption (for example, in the National Institute for Health and Care Excellence (NICE)’s appraisals of nusinersen [8] and risdiplam [9] for spinal muscular atrophy), there has been much less empirical evidence on how carers’ HRQoL changes after patient death. Landfeldt and Sandhu suggested that the inclusion of a bereavement effect on HRQoL could “solve” the carer QALY trap, if the effect was sufficiently large and sustained over time [10]. Without a bereavement effect, increased survival leads to increased carer disutility; however, if there is a sustained bereavement effect then carer disutility persists beyond the death of the patient and increased survival no longer leads to increased carer disutility.

If we believe that economic evaluations should reflect all important differences between costs and/or outcomes, and that carers’ HRQoL should be included, it is logical that time horizons should be extended to reflect all differences in carers’ HRQoL outcomes. This would mean that any bereavement effects should be included until such a time that there is no longer any difference—either the carers’ HRQoL has returned to the general-population level or has stabilised such that bereaved carers’ HRQoL at time *t* + 1 is equivalent to bereaved carers’ HRQoL at time *t* (after accounting for any expected effects such as aging).

However, if we determine that bereavement affects the HRQoL of the patient’s network, then it would seem inequitable and inconsistent to only include this bereavement effect for carers if it also affects people who cared about but did not report providing unpaid care to the deceased. By this argument, a bereavement effect should be included for any economic evaluation in which people die, and (assuming it is consistent over time), would only differ between technologies due to patients dying at different times, and discounting meaning that future health outcomes are valued less.

It is plausible that carers and non-carers do experience bereavement differently. Schulz et al. summarise three hypotheses that have been proposed for the effect of bereavement on caregiving:

1. Cumulative stress: the combined effects of caregiving stress and death lead to worse outcomes after bereavement for carers;
2. Stress reduction perspective: death reduces caregiving stressors, so carers have better outcomes than non-carers after bereavement;
3. Anticipatory grief: carers are more likely to anticipate and experience grief before the person dies, leading to worse outcomes pre-bereavement but reduced distress after bereavement [11].

Whilst previous longitudinal studies have explored the relationship between bereavement and measures of grief [12–14] and depression [15], there is a lack of evidence studying the relationship between bereavement and HRQoL, as measured by utility instruments for use in economic evaluation. We therefore aim to answer the following research questions:

1. How does carers’ HRQoL change after the person they care for dies—is there a bereavement effect, and is this positive or negative?
2. Does carers’ HRQoL become comparable with the general population after the person they care for dies—and if so, after what period?
3. Is there an effect on HRQoL of bereavement for the network of people who do not provide care for the patient—and how does this compare to the bereavement effect for carers?

## Methods

### Data

We used observational panel data from Understanding Society (United Kingdom Household Longitudinal Survey) waves 1–12 [16]. This is a household survey which asks all members of included households to provide the same information in each annual wave. The design of UKHLS means that it captures the scenarios in which some people care for household members and some people do not, and that some people in both groups are bereaved when the household member (who they do or do not care for) dies. Data are collected in each wave on who lives within each household and their relationship to each other, and whether anyone who previously lived within the household has died. All household members aged over 16 years complete surveys on a range of topics including a caring module, which identifies whether they provide care for another household member, and whom they provide care for. Household members who have moved out of the family home and into an institution such as a care home are still classed as household members and included in the survey [17]. Respondents also complete the Short-Form 12, which can be used to calculate the Short-Form 6D [18]. Demographic information such as age and sex are routinely collected.

### Analysis

We wanted to know how HRQoL changes when a person is bereaved and so we needed to analyse their HRQoL before and after bereavement. However, we know that HRQoL changes over time due to aging and other factors and so we wanted to compare the change in HRQoL following bereavement with how their HRQoL would have changed if that family member had not died. We were therefore interested in the “Difference-in-Differences” between people who are bereaved and people who are not bereaved.

If we had only two data points for each group (before and after bereavement for the bereaved group, and the same time points for the non-bereaved group), we could calculate the average HRQoL for each group at each time point, calculate the difference in HRQoL for each group, and calculate the difference in differences (or “four averages and three subtractions” [19], illustrated in Appendix Fig. S1, see electronic supplementary material [ESM]). However, we had up to 12 time points for each person and bereavement can occur at any of these time points (differential timing) and we were interested in analysing the immediate and longer-term effect on HRQoL, and so we used an event study [20].

We used the method described by Callaway and Sant’Anna [21] to estimate the dynamic (varying over time) average treatment effect on the treated (ATT). This uses as a reference period the most recent time period when untreated potential outcomes are observed for units in each group. The comparison group is all “never-treated” units at the same time point. The effects are then aggregated to allow identification of the event time effects, to produce an event study wherein treatment effects can vary by the time since the event.

We used outcome regression (OR) to control for observed confounders in estimating the effect of bereavement on HRQoL, and inverse probability weighting (IPW) to balance the characteristics in the groups who are and are not bereaved. Combining OR and IPW gave us a doubly robust estimand which required only one of the OR or IPW approaches to be correctly specified. We used the csdid community-contributed command in Stata [22].

### Assumptions

We made the following assumptions:

1. Irreversibility of treatment: since the ‘treatment’ here is the death of a household member, it is surely irreversible.
2. Stable unit treatment value assumption: a treated unit cannot impact a control unit (since we account for all within-household bereavements, any person who is bereaved by the death of one household member is included in the treated group).
3. Limited treatment anticipation: people do not choose for their household member to die, and do not change their behaviour if they become aware this will happen.
4. Conditional parallel trends: people who become bereaved and those who do not become bereaved would otherwise have the same HRQoL trajectory, conditional on their age, sex, educational qualification, household income, and presence of a long-standing illness/health condition (because these are known to influence HRQoL and trajectories [23]). People cannot select into bereavement—for context, euthanasia/assisted dying is not legal in the UK [24] and so we do not believe that people whose outcomes would be differentially affected by bereavement are able to differentially select into becoming bereaved. Household members requiring care may move into a care home or go into hospital but are still classed as household members and remain in the study—people therefore did not exit the study when either the caregiving burden increased or the household member approached death, which may have violated the conditional parallel trends assumption. We recognise that some households may be subject to higher mortality rates and worse health, in which case we may expect that people whose household members die are likely to experience worse health themselves, and so we conditioned on socioeconomic variables (education and income) as well as presence of long-standing illness/health conditions.

### Populations and Comparisons

We defined a carer as answering “Yes” to the question: “Is there anyone living with you who is sick, disabled or elderly whom you look after or give special help to (for example, a sick, disabled or elderly relative, husband, wife or friend etc.)?” in any wave of UKHLS. This population therefore contained people who went on to become carers in the future but were not currently carers at some timepoints—we believe this is appropriate given that carers often take a number of years to identify as carers [25] and so there may be a delay in carers responding “Yes” to this question. In a scenario analysis, we considered a subgroup of this population and analysed data only from people who had started providing care at each timepoint. This population also contained people who previously answered that they were providing care, and now answer that they are not—these people were included because of the potential for them to adapt to their role and no longer recognise themselves as carers, or where the person they care for now lives in a care home. People who never answered “Yes” to this question were classed as not carers.

We defined a household member as ‘bereaved’ in wave *w* where the residence of another household member in wave *w* was recorded as ‘dead’. We do not know the exact timing of the death, only that it occurred between waves *w − *1 and *w*. Where someone has been bereaved more than once, we included only the first bereavement as an event and continued to include the bereaved person’s outcomes in the analysis, to reflect the reality that carers and non-carers may be bereaved more than once. People who do not have any household member recorded as ‘dead’ in any wave were classed as not bereaved.

Where someone was defined as a carer and as bereaved, we only classified them as a “carer who experiences bereavement” if they mentioned that they provide care to the person who died at any wave before that person died.

We compared the following groups:

1. Carers who experience bereavement versus carers who do not experience bereavement (to understand how bereaved carers’ HRQoL compares with that of carers who continue caring);
2. Carers who experience bereavement versus people who are not carers and who do not experience bereavement (to understand how bereaved carers’ HRQoL compares with that of a population who are not carers);
3. People who are not carers and who experience bereavement versus people who are not carers and who do not experience bereavement.

We compared the mean and confidence intervals for the estimates from groups 1 and 3 to understand if the effect sizes differ for carers and non-carers.

In scenario analysis, we compared bereaved carers to “not yet” bereaved carers (carers who become bereaved in later waves) and bereaved non-carers to “not yet” bereaved non-carers. This was to address concern that the not-bereaved populations were not similar enough to the bereaved population.

To understand the mechanism behind any changes in HRQoL, we examined the proportion of patients reporting each response to the seven SF-12 questions that are used in calculating the SF-6D, before and after bereavement. We did this separately for bereaved carers and bereaved non-carers.

We performed scenario analyses based on the sex of the bereaved, relationship to the deceased, and age of the deceased at death to explore heterogeneity within the bereavement effect.

## Results

### First Observed Characteristics

Table 1 shows the characteristics for carers and non-carers, who do and do not experience bereavement at first observation in the dataset. We note that these are not the baseline characteristics used in the analysis, where baseline is the period before treatment (bereavement) for the ‘treated’ (bereaved) population, and for the control group is the pre-period for the comparison at each given time. For example, for people who become bereaved at wave 2, the control group is people who have observations at both wave 1 and wave 2 and the baseline characteristics are those reported at wave 1. First observation characteristics are presented here to allow comparison of the populations and to identify characteristics which may differ at entry, and which may be expected to influence HRQoL trajectories.

**Table 1:** Baseline characteristics at first observation, unadjusted. Table presents number and (% percentage) or mean and (standard error)

	Bereaved and carer status				
	Bereaved carer	Non-bereaved carer	Bereaved non-carer	Non-bereaved non-carer	Total
*N*	1447 (2.6%)	8846 (15.9%)	721 (1.3%)	44,731 (80.2%)	55,745 (100.0%)
Age	56.576 (18.104)	45.412 (18.803)	52.165 (20.765)	39.235 (17.225)	40.832 (17.937)
Sex					
Female	891 (61.6%)	4940 (55.8%)	430 (59.6%)	24,071 (53.8%)	30,332 (54.4%)
Male	556 (38.4%)	3906 (44.2%)	291 (40.4%)	20,655 (46.2%)	25,408 (45.6%)
Bereaved relation to deceased					
Spouse/partner	869 (60.1%)	0 (0.0%)	475 (66.1%)	0 (0.0%)	1344 (62.0%)
Child	434 (30.0%)	0 (0.0%)	136 (18.9%)	0 (0.0%)	570 (26.3%)
Parent	20 (1.4%)	0 (0.0%)	41 (5.7%)	0 (0.0%)	61 (2.8%)
Sibling	22 (1.5%)	0 (0.0%)	11 (1.5%)	0 (0.0%)	33 (1.5%)
Other relative	7 (0.5%)	0 (0.0%)	5 (0.7%)	0 (0.0%)	12 (0.6%)
Non-relative	31 (2.1%)	0 (0.0%)	30 (4.2%)	0 (0.0%)	61 (2.8%)
Grandchild	63 (4.4%)	0 (0.0%)	21 (2.9%)	0 (0.0%)	84 (3.9%)
Grandparent	1 (0.1%)	0 (0.0%)	0 (0.0%)	0 (0.0%)	1 (0.0%)
Educational achievements					
None	458 (38.7%)	2290 (30.8%)	211 (35.2%)	7592 (20.0%)	10,551 (22.4%)
Secondary school	446 (37.7%)	3353 (45.1%)	262 (43.7%)	18,120 (47.7%)	22,181 (47.0%)
Post-secondary	62 (5.2%)	515 (6.9%)	31 (5.2%)	2959 (7.8%)	3567 (7.6%)
Undergrad degree	105 (8.9%)	669 (9.0%)	50 (8.3%)	5162 (13.6%)	5986 (12.7%)
Postgrad degree	77 (6.5%)	471 (6.3%)	29 (4.8%)	3575 (9.4%)	4152 (8.8%)
Medical/nursing qualification	34 (2.9%)	134 (1.8%)	16 (2.7%)	548 (1.4%)	732 (1.6%)
Income (log)	7.061 (0.600)	7.030 (0.594)	7.123 (0.620)	7.225 (0.617)	7.188 (0.618)
Long-standing illness					
No	808 (55.8%)	5133 (58.0%)	453 (62.8%)	32,644 (73.0%)	39,038 (70.1%)
Yes	639 (44.2%)	3710 (42.0%)	268 (37.2%)	12,064 (27.0%)	16,681 (29.9%)
Number of people cared for					
0	0 (0.0%)	159 (1.8%)	721 (100.0%)	44,731 (100.0%)	45,611 (81.8%)
1	1239 (85.6%)	7281 (82.3%)	0 (0.0%)	0 (0.0%)	8520 (15.3%)
2	171 (11.8%)	1072 (12.1%)	0 (0.0%)	0 (0.0%)	1243 (2.2%)
3	27 (1.9%)	243 (2.7%)	0 (0.0%)	0 (0.0%)	270 (0.5%)
4	9 (0.6%)	60 (0.7%)	0 (0.0%)	0 (0.0%)	69 (0.1%)
5	1 (0.1%)	17 (0.2%)	0 (0.0%)	0 (0.0%)	18 (0.0%)
6	0 (0.0%)	11 (0.1%)	0 (0.0%)	0 (0.0%)	11 (0.0%)
7	0 (0.0%)	2 (0.0%)	0 (0.0%)	0 (0.0%)	2 (0.0%)
8	0 (0.0%)	1 (0.0%)	0 (0.0%)	0 (0.0%)	1 (0.0%)
Bereaved SF6D	0.779 (0.137)	0.770 (0.142)	0.798 (0.131)	0.812 (0.123)	0.804 (0.127)
Age of person cared for	66.392 (12.322)	56.976 (16.887)	0.0 (0.0)	0.0 (0.0)	58.251 (16.655)
Sex of person cared for					
Female	84 (37.8%)	782 (55.1%)	0 (0.0%)	0 (0.0%)	866 (52.8%)
Male	138 (62.2%)	636 (44.9%)	0 (0.0%)	0 (0.0%)	774 (47.2%)
SF6D of person cared for	0.770 (0.131)	0.732 (0.151)	0.0 (0.0)	0.0 (0.0)	0.737 (0.149)

In comparing bereaved carers with non-bereaved carers, we note that those who are bereaved are on average older, more likely to be female, more likely to have no educational qualifications, have on average lower income, but have similar baseline HRQoL scores. In comparing bereaved non-carers with non-bereaved non-carers, we note that those who are bereaved are on average older, more likely to be female, and more likely to have no educational qualifications. Income, presence of long-standing illness and initial HRQoL scores were similar. In comparing bereaved carers with bereaved non-carers, we note that carers are older, have a lower income, more likely to have a long-standing illness, but have a similar sex distribution and baseline HRQoL score.

As there were differences in age, sex, education, income and presence of a long-standing illness, and these may influence future health and HRQoL, we controlled for these using IPW and OR. We assume that the parallel trend assumption holds, conditional on these variables.

Most people are bereaved by the death of a spouse, but this is slightly less likely for carers where the proportion whose parent died is higher. A small number of people were bereaved by the death of a child, and this was less likely for carers than non-carers.

Characteristics for the cared-for person are also shown where the carer only provides care to one person (the majority of carers). The cared-for people who died in the dataset were older at first observation (as expected with bereaved carers being older and most carers being bereaved by the death of spouse) and a higher proportion were male (as expected with most carers being female spouses). Surprisingly, the cared-for who later died had higher HRQoL scores at first observations. However, these tended to decline rapidly in the period immediately before death, as shown in Appendix Fig. S2 in the ESM.

The last recorded characteristics of the deceased are tabulated by whether they were cared for in Appendix Table S1 (see ESM). People who died after being cared for were on average older and had much lower HRQoL scores and notably worse physical health outcomes. The proportion of the deceased who were recorded as previously diagnosed with specific health conditions is presented graphically in Appendix Fig. S3 (see ESM), where we see higher prevalence of most diseases in the population who were cared for before they died.

**Fig. 1:**
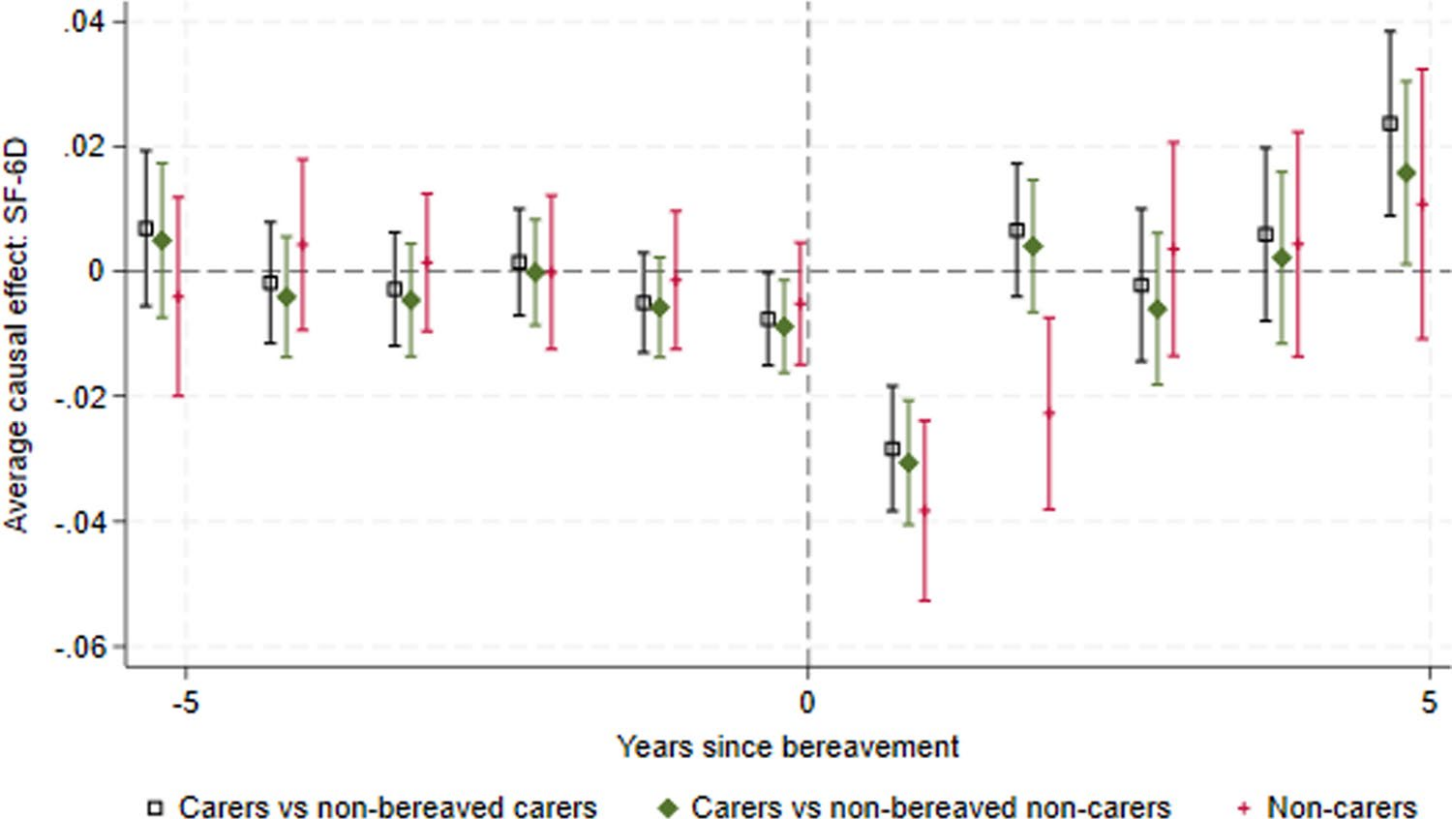
Effect of bereavement on SF-6D. Carers 1 = bereaved carers vs non-bereaved carers. Carers 2 = bereaved carers vs non-bereaved non-carers (household members who did not report providing care)

Figure 1 presents the ATT for the SF-6D for each period before and after the event, where the event is bereavement (Appendix Fig. S4 in the ESM presents the full study duration). The plot presents three comparisons: bereaved carers versus non-bereaved carers, bereaved carers versus non-bereaved non-carers, and non-carers versus non-bereaved non-carers. Unadjusted results were also undertaken and can be found in Appendix Fig. S5 (see ESM). The findings were similar regardless of whether adjustment was included.

### Prior to the Event

There is some variation in the ATT before period 0, but the confidence intervals tend to span zero (no causal effect), suggesting that the parallel trends assumption holds prior to bereavement. The ATT for both bereaved carers compared with non-bereaved carers and bereaved carers compared with non-bereaved non-carers comparisons becomes negative at period 0—this may reflect anticipatory grief (grief experienced before the death of the cared-for, due to realising what is about to happen, preparing for life without the dying person, and feelings of freedom or isolation [26] ) or it may reflect the known HRQoL loss associated with caregiving as the proportion of carers caring is highest at this timepoint [27, 28].

The mean SF-6D loss in the period immediately before death is − 0.00762 (95% CI − 0.0151 to − 0.00173) for carers compared with non-bereaved carers and is slightly smaller and not statistically significant for non-carers (− 0.00518, 95% CI − 0.0149 to 0.00459). The caregiving burden also increases in the period immediately before death, with a higher proportion of carers reporting providing care in this period (see Appendix Fig. S6 in ESM). It is therefore unclear whether the decrease in SF-6D in this period is due to the increased caregiving burden or anticipatory grief. When we consider only co-resident carers and non-carers bereaved by the death of the same person (e.g., an elderly woman caring for her husband whose adult child lives in the same household and does not provide care), the SF-6D losses are more similar and confidence intervals overlap (− 0.0240, 95% CI − 0.0489 to 0.008 for non-carers and − 0.0312, 95% CI − 0.0583 to − 0.00424 for carers). This supports the theory that the HRQoL loss immediately before death is due at least partly to anticipatory grief since co-resident non-carers would also experience anticipatory grief but not the caregiving burden.

### Short-Term Bereavement Effects

Carers (both comparisons) and non-carers experience a significant loss in HRQoL in the period (period 1) immediately following bereavement: carers: − 0.0283 (95% CI − 0.0432 to − 0.0134), non-carers: − 0.0383 (95% CI − 0.0527 to − 0.0239). The confidence intervals overlap, suggesting that the initial bereavement effect is not statistically significantly different for carers and non-carers. The losses are relatively small when compared with the disutilities associated with caring [27] or with adverse events, for example arising from cancer treatments [29], suggesting that the effect of bereavement on HRQoL is relatively minor. In a naïve comparison of SF-6D scores for the combined population of carers and non-carers before and after bereavement, the HRQoL loss is − 0.0327 (95% CI − 0.0395 to − 0.0260).

We explored the impact of using the General Health Questionnaire (GHQ); again, we found an anticipatory effect for carers, a statistically significant effect in the year immediately following bereavement for carers and non-carers, and that this effect was numerically greater for non-carers than carers. While the ATT for the carers groups is close to zero 2 years after bereavement, the bereavement effect for non-carers appears to extend into year 2. The GHQ, a broader measure of wellbeing, showed similar scores for carers and non-carers 2 years after bereavement, with all CIs spanning zero (see Appendix Fig. S7 in the ESM). This suggests that there may be a difference in the duration of the bereavement effect for carers and non-carers but that this is sensitive to the instrument used. The SF-12 Mental Component Summary (MCS) tells a similar story, with all groups having a similar and statistically significant decrease in the year immediately following bereavement, and the non-carers group having an effect extending into year 2 (see Appendix Fig. S8 in the ESM). The SF-12 Physical Component Summary (PCS) suggests that physical health improves after bereavement (see Appendix Fig. S9 in the ESM), but we urge caution in interpreting this as the scoring system for PCS has negative coefficients for the questions related to mental health meaning that a decrease in mental health leads to an improvement in PCS, ceteris paribus [30].

### Long-Term Bereavement Effects

The results suggest that there is no sustained effect on HRQoL beyond 2 years after bereavement. The CIs for the ATTs for all groups tend to span zero, but we note that there is some variation likely due to low numbers of observations as the years since bereavement increases. It is notable that the bereaved carers versus non-bereaved carers mean is generally above zero and the bereaved carers versus non-bereaved non-carers mean below zero until year 5—this suggests that bereaved carers’ HRQoL may increase compared with providing ongoing care for a surviving patient.

### Comparison of Bereavement Effects for Carers and Non-Carers

We compared the effect sizes at year 1 after the event from the Group 1 and Group 3 Difference-in-Differences where the confidence intervals (CI) overlap. At year 2, the effect for carers is non-significant: 0.00659 (95% CI − 0.00405 to 0.0172) and for non-carers is − 0.0227 (95% CI − 0.0380 to − 0.00741). We concluded that there are no statistically significant differences between the bereavement effects for carers and non-carers in the first year, and that there is a trend for a prolonged bereavement effect for non-carers in year 2 but the confidence intervals overlap.

### Mechanism/Domains of SF-6D

**Fig. 2:**
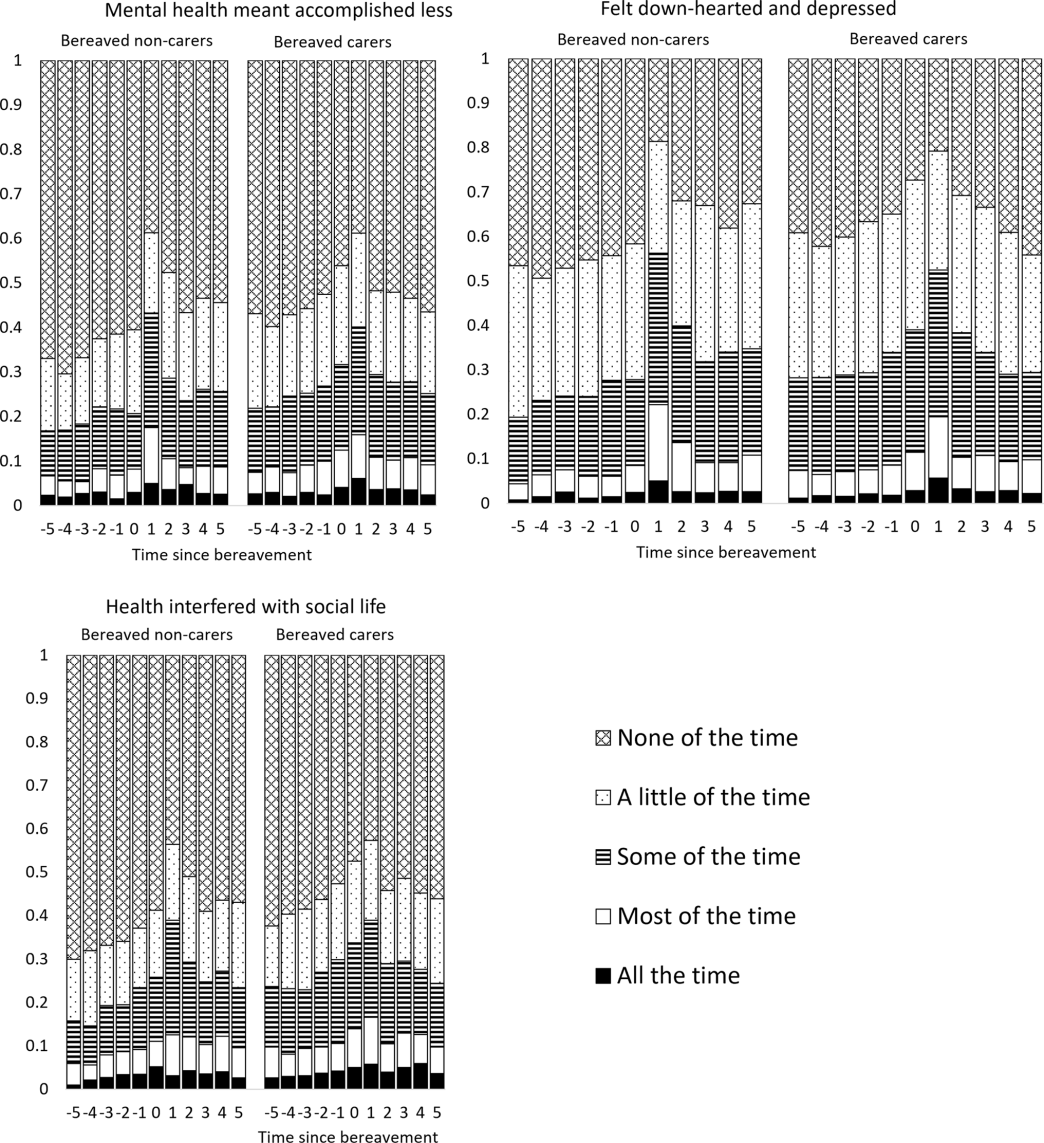
Responses to SF-6D domains for mental health, feeling downhearted and depressed, and social life, by time to bereavement for bereaved non-carers and bereaved carers

The questions that seem to exhibit the biggest change after bereavement are 4a (emotional problems have caused problems accomplishing work or other regular activities), 6c (feeling downhearted or depressed) and 7 (physical health or emotional problems interfered with social life). The proportion of people answering in each category to these questions before and after bereavement are shown in Fig. 2.

**Fig. 3:**
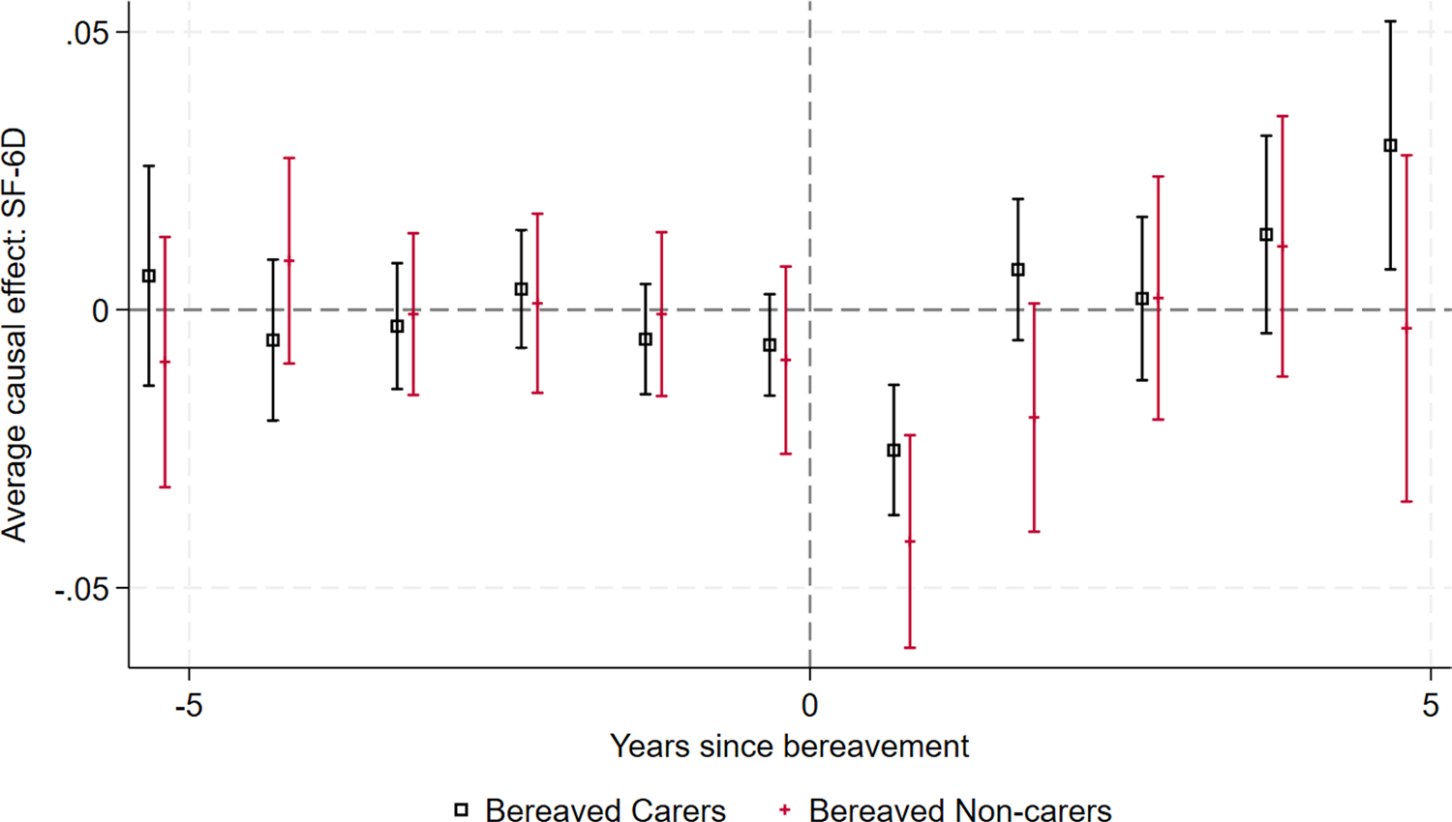
Effect of bereavement on SF-6D, comparing bereaved carers with not-yet-bereaved carers, and comparing bereaved carers with not-yet-bereaved non-carers

For each of these domains, in the period immediately following bereavement there is a reduction in the proportion of patients reporting the higher (better) scores and an increase in the proportion reporting lower scores. For bereaved carers, the effect appears to last only 1 year, whereas for non-carers the graphs suggest it may take 2 years after the event for the proportions to return to the pre-event trend. This corresponds to the non-significant negative ATT in period 2 for bereaved non-carers in Fig. 3, compared with the non-significant positive ATT in period 2 for bereaved carers in Fig. 1.

There appears to be almost no change in 3b (physical health limiting kind of work), 5 (pain interfering with work), 6b (having a lot of energy) and only a small change in 2a (health limiting moderate activities) (see Appendix Fig. S10 in the ESM).

It appears to be the mental health/emotional impact of bereavement that is driving the change in HRQoL rather than physical health.

### Comparison With Not-Yet-Bereaved

For carers, there is no statistically significant anticipation effect (− 0.00635, 95% CI − 0.0155 to 0.00278) and the short-term bereavement effect is − 0.0252 (95% CI − 0.0369 to − 0.0135) in the first year. For non-carers, the short-term bereavement effect is − 0.0416 (95% CI − 0.0608 to − 0.0225), and the effect is not statistically significant beyond the first year. The trends and effect sizes are similar to the never-treated comparison, but the sample sizes are smaller and so the confidence intervals are wider. The graph is presented in Fig. 3.

### Subgroup Analysis

#### Alternative Carer Populations

In the population where carers must have reported providing care before their baseline for the control group, the trends and effects sizes were similar to the base case, but the confidence interval increased, due to the reduced sample size (Appendix Fig. S11, see ESM). The same was true for the subgroup of carers who provided care only to one household member (Appendix Fig. S12).

#### Sex

**Fig. 4:**
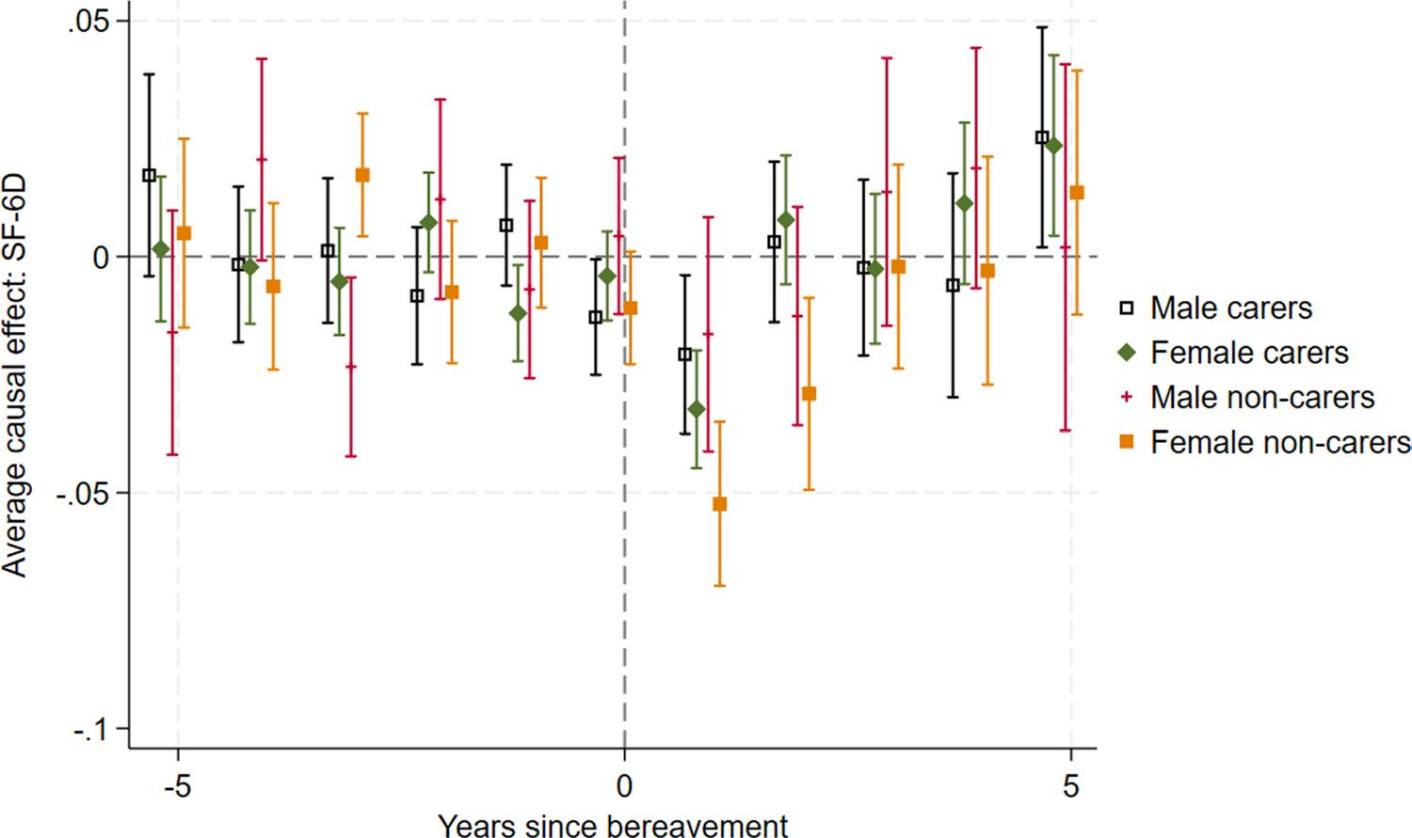
Effect of bereavement on SF-6D for subgroups based on sex and caring status

In the subgroup analysis in Fig. 4, it is notable that female non-carers have the greatest HRQoL loss in the first two periods after bereavement. Male non-carers have the smallest HRQoL loss initially. Male carers appear to experience the greatest anticipation effect. This may suggest that males and females are affected differently by bereavement, and that the impact of caring on the bereavement effect may differ by sex. However, the confidence intervals tend to overlap, suggesting these differences are not statistically significant.

#### Relationship

**Fig. 5:**
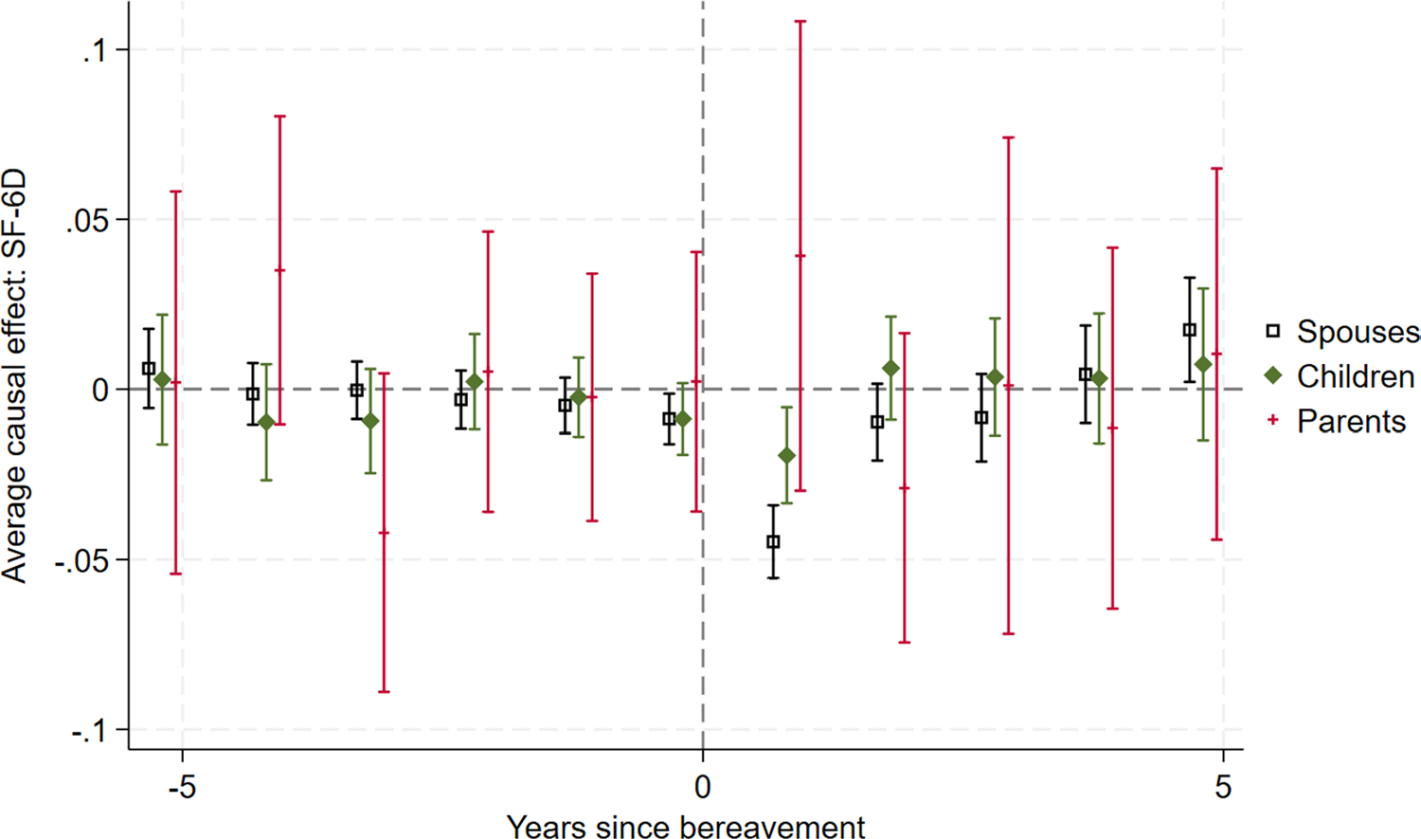
Effect of bereavement on SF-6D for subgroups based on the bereaved person’s relationship to the deceased

When we examine the relationship of the bereaved to the deceased (including carers and non-carers combined), the HRQoL loss appears to be largest for spouses, while the effects for (adult) children whose parents die are smaller (Fig. 5). The HRQoL change for parents whose children die are not significant, due to the much smaller sample size (*n* = 47) and outcomes for this group are highly uncertain. We found similar results using the GHQ (see Appendix Fig. S13 in the ESM). The last recorded characteristics of the deceased person are tabulated by relationship in Appendix Table S2 (see ESM).

#### Age at Death

**Fig. 6:**
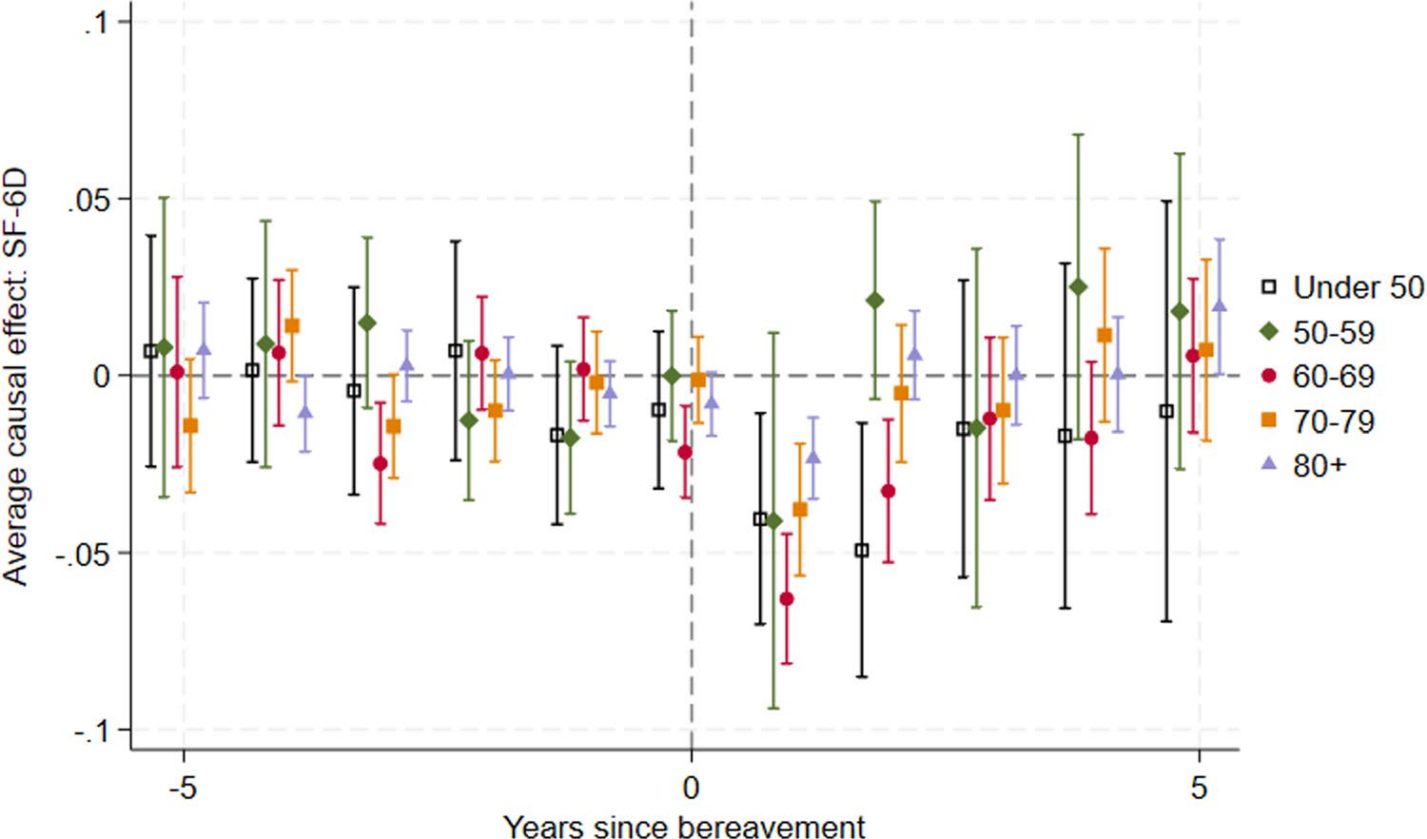
Effect of bereavement on SF-6D for subgroups based on the deceased person’s age at death

In Fig. 6, we show the effect of the age at which the deceased died on the bereaved person’s HRQoL changes. The point estimates for the effect sizes generally appear to be smaller when people die aged 80+ years than when they die aged 60–79 years, but the confidence intervals generally overlap. The effect size for when people die aged under 50 years or 50–59 years are similar to each other and the other age groups in the first year after bereavement. In the second year after bereavement, the effect size appears bigger where people died aged under 50 years (but still less than −0.05 and not statistically significantly different to where people died aged 60–69 years) and smaller where people died aged 50–59 years. By 3 years after bereavement, the effect sizes appear similar regardless of the age at which the deceased died. We note that the confidence intervals are wide for the younger ages as there are fewer deaths at these ages (see Appendix Fig. S14 in the ESM).

## Discussion

### Main Findings

We find that there is a bereavement effect for carers and non-carers in the form of a similarly sized small decrease in HRQoL in the first year following bereavement.

After the first year, the HRQoL of bereaved carers (and potentially after the second year for non-carers depending on HRQoL measure) becomes comparable to the non-bereaved population, suggesting there is no long-term bereavement effect. This is consistent with evidence suggesting bereavement effects are short-lived [13, 31] and typically resolve within 2 years [32]. We recognise that some people will experience complicated grief which may be more intense and last for longer [33, 34], but our study focusses on average effects, as would be applied in economic evaluations.

Our analysis of the domains of SF6D and the SF-12 MCS and PCS suggests that the short-term HRQoL loss is due to changes in mental health, emotions, and social functioning following bereavement, and not due to changes in physical health. This is consistent with bereavement research which has focussed on depression and anxiety [12]. It is possible that other measures of HRQoL/wellbeing which place greater importance on mental health may detect a larger effect size.

Our analysis suggested that the duration of the bereavement effect may be longer for non-carers than carers when using SF-6D. There are several potential explanations for why the duration of the bereavement effect may be shorter for carers than non-carers; carers may be more prepared for the death of the patient, and low preparedness is associated with more complicated grief and worse symptoms [35], or caregivers may feel that the death of the patient was a relief to themselves due to protracted and stressful caregiving [31], or carers may experience anticipatory grief in preparing themselves for the death of the patient [36]. However, other research has suggested that anticipatory grief does not alleviate bereavement outcomes [12] and a cross-sectional analysis suggested that while bereaved people have lower wellbeing than non-bereaved people, there were no differences between bereaved carers’ and bereaved non-carers’ wellbeing [37].

### Implications

Our results suggest that there is a short-term negative impact of bereavement on HRQoL for both carers and non-carers, and that there is no evidence to suggest these effects differ between carers and non-carers in the first year. Longer term, carers’ HRQoL returns to general population levels after the person they care for dies. Our results do not suggest that the impact of bereavement differs substantially when adults die at different ages (we note our sample included very few children who died). Therefore, in economic evaluations of life-extending interventions, the only differential impact on bereavement would be due to discounting (since all modelled patients in both arms would die in a model with a lifetime horizon)—and given the small size of the bereavement effect, the differences would be negligible. Based on our findings, including a bereavement effect would not solve the carer QALY trap. We propose, therefore, that carers’ HRQoL should be included in economic evaluation only up until the point at which the patient dies.

If we believe that utility measures accurately reflect carers’ preferences, then we recognise this may lead to the unpalatable finding that interventions that extend patient survival may lead to a QALY loss for carers. However, we believe that this is an accurate representation of the current evidence and the issue with interpreting the results is because of the way that carer HRQoL is currently included in economic models. As noted by Tilford and Tarlan, such analyses do not capture altruism where carers place greater value on improving patient survival than they do on relieving their own caregiving burden [38]. As noted by the economist Nancy Folbre in discussing how care can be captured in economics (page 102) [39]:

“All seemingly non-economic motivations for providing care services can be subsumed under the rubric of utility maximization. Individuals can derive utility either directly from the well-being of others or indirectly from ‘doing the right thing’.”

Cost-utility analysis relies on the assumptions that people are rationally self-interested, but this is not necessarily the case for carers. Eva Kittay, in her essays on caring for a dependent child, describes the “transparent self”, a self that is so passionately interested in the wellbeing of another person that they see first the needs of the other (page 52) [40]. Consistent with Tilford and Tarlan’s suggestion that the scope for estimating QALYs for carers requires a comparison not between carers and non-carers but between carers who care for a patient and bereaved carers [38], Kittay describes that the worst moment of her life was not being told of her child’s diagnosis, but the moment she feared that her child would die (page 164).

If we want to avoid the scenario that extending patient survival improves carers’ HRQoL, then we should not dismiss our finding. Instead, analysts and policy makers should question the purpose and methods for including carer HRQoL in economic models. Furthermore, policy makers should consider how they can support carers to improve their HRQoL while the person they care for is alive.

### Comparison with Literature

There is little evidence to date exploring the relationship between bereavement and HRQoL, but we identified one study by Song et al. (2010) from the US that compared the Health Utilities Index (HUI) Mark 3 scores of 233 couples who were bereaved by the death of a child with 229 couples who were not bereaved by the death of a child, using stratified matching [41]. They found bereaved couples’ HRQoL was statistically significantly worse, by 0.04 units. When including information on cause of child death, HRQoL was only statistically significantly worse for parents whose child died a violent death (and not due to illness or infant death). We note that our sample size for this population was smaller, we used a different HRQoL measure, the causes of death may differ (we do not have information on cause of death), our populations differ (only 12 of the 47 deceased children in our sample were aged under 16 years) and our methods are different as Difference-in-Differences uses information on HRQoL before as well as after bereavement. Song et al. (2010) did not control for HRQoL before bereavement and used only one wave of data.

Camacho et al. surveyed EQ-5D scores for 256 parents who experienced a perinatal death [42] and compared them with general population EQ-5D scores. They found a significant difference in EQ-5D in the 12 months since perinatal death (26% in months 0–6 and 19% in months 7–12). Between 1 and 10 years, there was still a significant difference in EQ-5D, but this was somewhat smaller (ranging from 9% to 14%) and did not display a clear trend with time. After 10 years, the differences were not significant. They suggested that the loss in HRQoL was driven by the changes in the anxiety/depression domain. The relationship between HRQoL and time since bereavement and the domains affected are consistent with our study. However, we note that there are some differences in the populations, particularly that stillbirths would not be included in UKHLS and bereaved parents represent a small proportion of our sample. There are also differences in the study design, with Camacho et al. noting the limitations of their cross-sectional study.

Song et al. surveyed the EQ-5D of 353 bereaved family members of patients with terminal cancer 2–6 months after the death of the patient and compared them with 353 controls using propensity score matching [43]. They found bereaved family members had statistically significantly worse EQ-5D than controls (effect size − 0.05) and that this was primarily due to worse scores in the anxiety/depression domain. Within the bereaved family members, males and people with higher incomes had higher EQ-5D and other factors had no statistically significant effect. Characteristics related to caregiving roles were not statistically significant. This is consistent with our finding that bereavement has a short-term effect on HRQoL, and that this effect does not differ between carers and non-carers.

Our finding that spousal carers have worse bereavement outcomes than adult children is also consistent with the literature [44]. Comparison for parents whose children have died is more difficult as much of the literature on child deaths focusses on young children, and those whose death is sudden and unexpected [45]. We note that there is a body of literature suggesting that the effects of child death can be long-lasting; Rogers et al. reported that bereaved parents had more depressive symptoms and worse well-being an average of 18 years after the death [46], Meert et al. observed higher rates of complicated grief in bereaved parents than the literature reported for bereaved spouses [47], and Maccallum et al. found differences in the shapes of grief trajectories for bereaved parents and spouses [15].

### Strengths

The strengths of our analysis lie in the large sample and number of years of data in UKHLS. Our analysis using Difference-in-Differences allows comparison of HRQoL changes in bereaved and non-bereaved populations (by considering data from before and after the bereavement), and we believe that conditional on the included variables, the parallel trends assumption holds. Using longitudinal data allows us to find stronger evidence of causality of bereavement on HRQoL, whereas cross-sectional data can only identify correlation.

### Limitations

A limitation of our dataset is that the cause of death is not recorded, and very limited information on disease is available from UKHLS. While our sample is generalisable to the UK population, we cannot make inferences for specific populations where there is unlikely to be data recorded in UKHLS (for example deaths due to rare events). It may not be appropriate to assume our findings apply to populations with substantially different baseline characteristics, for example, carers whose HRQoL while the person they care for is alive is substantially lower than in our study.

We also note that respondents self-report whether they provide care, and the interpretation of this may differ between respondents. Furthermore, people may start to provide care for a dying household member between the waves immediately before and after bereavement, leading to some overlap in our groups.

It is plausible that there is a greater HRQoL loss immediately following bereavement that our analysis does not detect because the survey is conducted annually, and we only record whether someone dies within 1 year. This may be relevant in economic evaluations that use a short (for example monthly) cycle length, if the acute impacts of bereavement differ between populations. However, the impact of this would be limited as it would be applied over a short time period.

A further limitation is that while the doubly robust method adjusts for differences in characteristics for comparison within groups, it does not account for the differences between groups. The comparison of the bereaved carers with either non-bereaved carers or non-bereaved non-carers makes the comparison group more like the bereaved carers, and the comparison for bereaved non-carers makes the non-bereaved non-carers more like bereaved non-carers. However, the differences between the bereaved carers and bereaved non-carers are not accounted for. This is potentially important given the differences between these populations and the differences in effects for different baseline characteristics.

While we explore the differences in bereavement effect by relationship, our sample size for bereaved parents is small and our findings therefore uncertain. More data would be required to definitively estimate the bereavement effect for this population. Such data would need to be gathered from targeted long-term follow-up of families of children with life-limiting diseases until after the child died, as child death is relatively rare in the general population. Until such data proves otherwise, we suggest that the conclusions from this research should apply—that is, that there is no evidence to support differentially including the effect of bereavement on HRQoL in economic evaluation.

## Conclusion

We conclude that there is a short-term HRQoL loss for bereavement, but that this effect does not differ between people who did and did not care for the deceased. We therefore conclude that economic evaluations should not include bereavement effects and should seek to find better ways to reflect carer HRQoL burden while the patient is alive.

## Supplementary Information

Below is the link to the electronic supplementary material.

Supplementary file1 (DOCX 1078 KB)

## Declarations

### Availability of data and materials

All data is freely available via the UK Data Service.
