# Supplementary material for: How Does Bereavement Affect the Health-Related Quality of Life of Household Members Who Do and Do Not Provide Unpaid Care? Difference-in-Differences Analyses Using the UK Household Longitudinal Survey
Source: Pharmacoeconomics. 2024 Dec 5;43(4):389–402. doi: 10.1007/s40273-024-01452-1 (PMC11929705; doi:10.1007/s40273-024-01452-1)
Supplement: Supplementary file 1 — Supplementary file1 (DOCX 1078 KB) [file 40273_2024_1452_MOESM1_ESM.docx]

**Appendix**

*Figure S1: visual example of 4 averages and 3 subtractions. In this example, there are four observations in each group at each time point.*

**
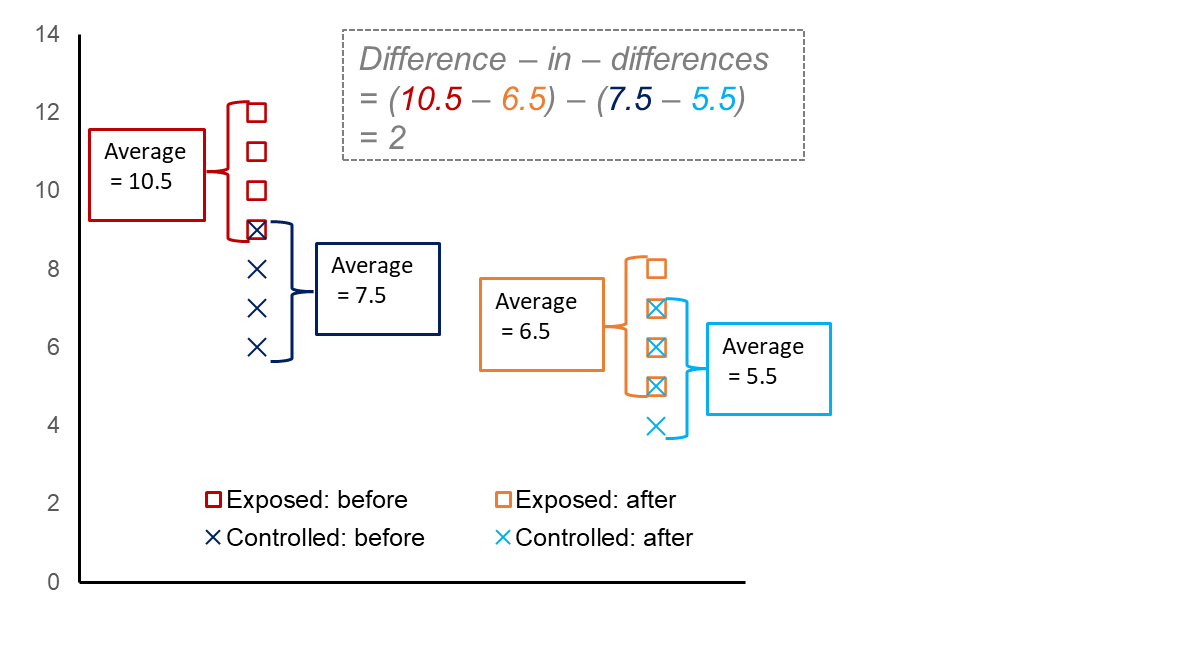
**

Table S1: last observed characteristics of deceased person, by whether they were cared for

|  | Bereaved and carer status | | |
| --- | --- | --- | --- |
|  | Bereaved carer | Bereaved non-carer | Total |
| N | 999 (64.6%) | 548 (35.4%) | 1,547 (100.0%) |
| Age | 75.356 (13.359) | 65.154 (17.427) | 71.752 (15.695) |
| Deceased under 16 |  |  |  |
| No | 999 (100.0%) | 548 (100.0%) | 1,547 (100.0%) |
| Sex, derived |  |  |  |
| Male | 573 (57.6%) | 333 (62.4%) | 906 (59.3%) |
| Female | 422 (42.4%) | 201 (37.6%) | 623 (40.7%) |
| Deceased's last SF-6D | 0.583 (0.125) | 0.751 (0.132) | 0.654 (0.152) |
| Deceased's last SF12 MCS | 43.170 (12.494) | 50.179 (10.223) | 46.130 (12.091) |
| Deceased's last SF12 PCS | 26.634 (10.332) | 42.216 (12.532) | 33.215 (13.680) |

SF6D, SF12 MCS and SF12 PCS only reported where deceased was aged over 16

Table S2: last observed characteristics of deceased person, by bereaved relationship to deceased

|  | Bereaved relation to deceased | | | | | | | | |
| --- | --- | --- | --- | --- | --- | --- | --- | --- | --- |
|  | Spouse/Partner | Child | Parent | Sibling | Other relative | Non-relative | Grandchild | Grandparent | Total |
| N | 1,057 (65.8%) | 386 (24.0%) | 47 (2.9%) | 22 (1.4%) | 5 (0.3%) | 40 (2.5%) | 49 (3.0%) | 1 (0.1%) | 1,607 (100.0%) |
| Age | 72.181 (11.968) | 74.351 (14.958) | 24.822 (17.726) | 56.636 (24.820) | 83.000 (5.831) | 59.846 (30.483) | 81.816 (7.960) | 16.000 (.) | 71.152 (16.250) |
| Deceased under 16 |  |  |  |  |  |  |  |  |  |
| No | 1,057 (100.0%) | 386 (100.0%) | 35 (74.5%) | 21 (95.5%) | 5 (100.0%) | 35 (87.5%) | 49 (100.0%) | 1 (100.0%) | 1,589 (98.9%) |
| Yes | 0 (0.0%) | 0 (0.0%) | 12 (25.5%) | 1 (4.5%) | 0 (0.0%) | 5 (12.5%) | 0 (0.0%) | 0 (0.0%) | 18 (1.1%) |
| Sex, derived |  |  |  |  |  |  |  |  |  |
| Male | 707 (66.9%) | 163 (42.3%) | 21 (63.6%) | 11 (52.4%) | 2 (40.0%) | 24 (70.6%) | 16 (32.7%) | 0 (0.0%) | 944 (59.6%) |
| Female | 350 (33.1%) | 222 (57.7%) | 12 (36.4%) | 10 (47.6%) | 3 (60.0%) | 10 (29.4%) | 33 (67.3%) | 1 (100.0%) | 641 (40.4%) |
| Deceased's last SF-6D | 0.669 (0.148) | 0.624 (0.157) | 0.723 (0.173) | 0.655 (0.152) | 0.585 (.) | 0.560 (0.154) | 0.588 (0.142) | 0.840 (.) | 0.658 (0.152) |
| Deceased's last SF12 MCS | 47.111 (11.610) | 44.040 (13.076) | 45.001 (12.843) | 47.967 (10.740) | 26.640 (.) | 39.483 (9.529) | 43.355 (10.692) | 51.670 (.) | 46.245 (11.973) |
| Deceased's last SF12 PCS | 34.092 (13.525) | 31.586 (13.723) | 47.793 (12.170) | 32.797 (12.435) | 31.480 (.) | 26.833 (12.680) | 28.897 (13.407) | 60.180 (.) | 33.648 (13.728) |

SF6D, SF12 MCS and SF12 PCS only reported where deceased was aged over 16

Figure S2: Average SF-6D of cared-for person


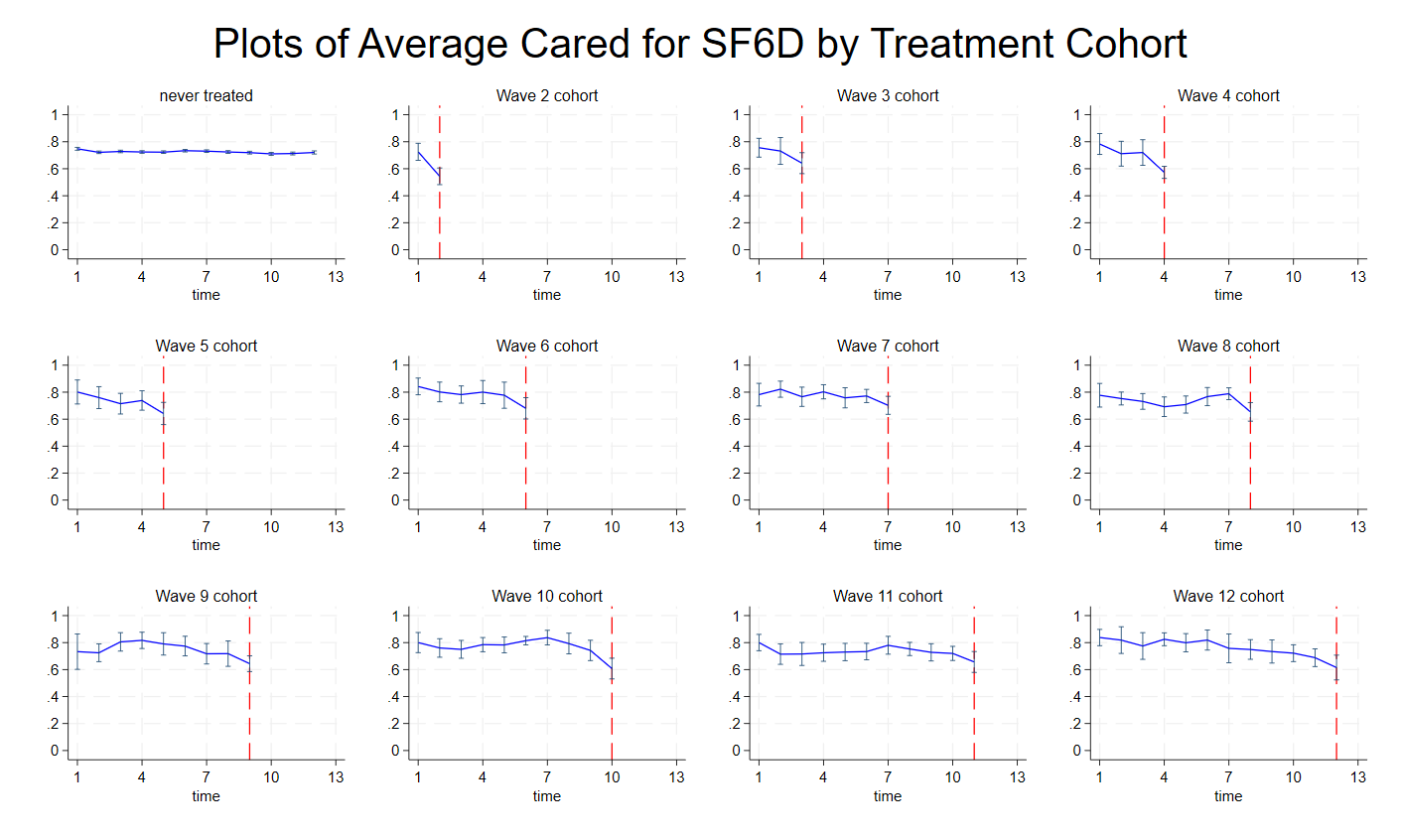


Figure S3: Percentage of the deceased with each recorded disease, by whether they were cared for before death

CHF: chronic heart failure, CHD: congenital heart disease, MI: myocardial infarction

Figure S4: Effect of bereavement on SF-6D. Carers 1 = bereaved carers vs. non-bereaved carers. Carers 2 = bereaved carers vs. non-bereaved non-carers (household members who did not report providing care). Conditional event study, full duration.


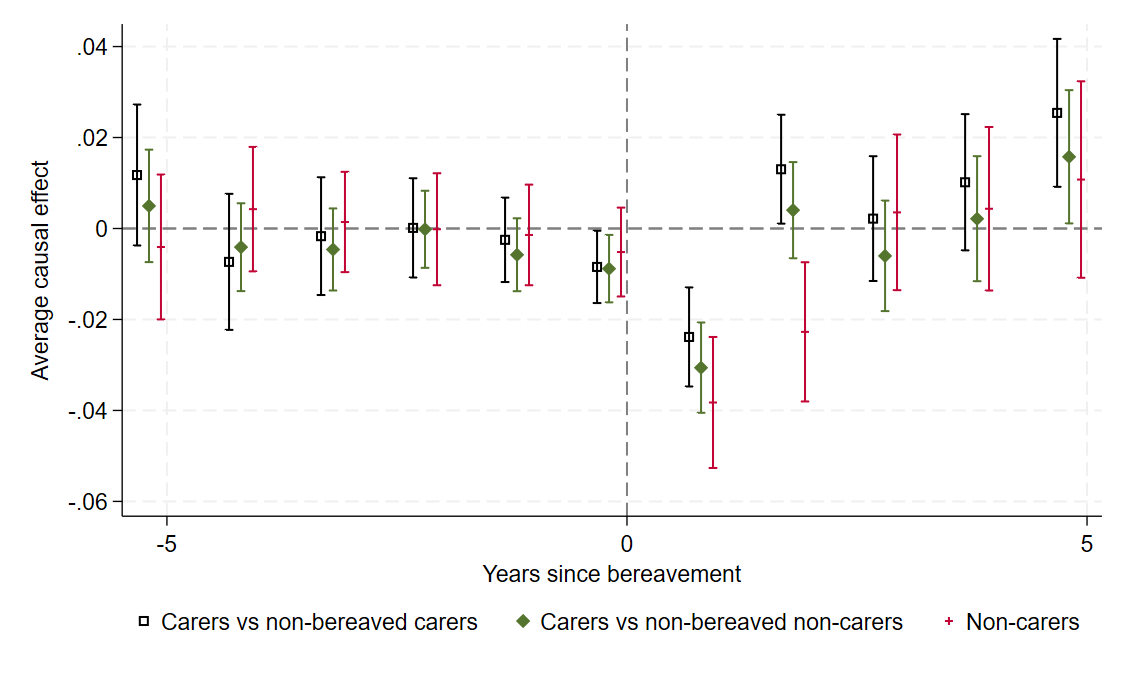


Figure S5: Effect of bereavement on SF-6D. Carers 1 = bereaved carers vs. non-bereaved carers. Carers 2 = bereaved carers vs. non-bereaved non-carers (household members who did not report providing care). Unconditional/unadjusted event study, full duration.


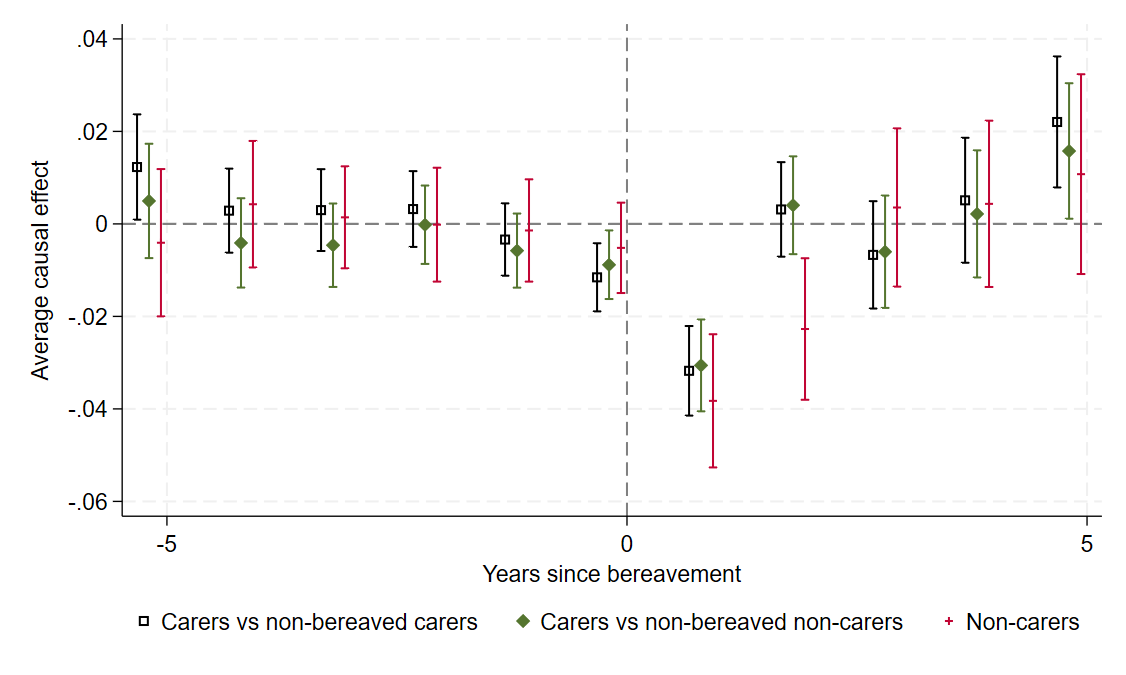


Figure S6: Average proportion of people caring in household.


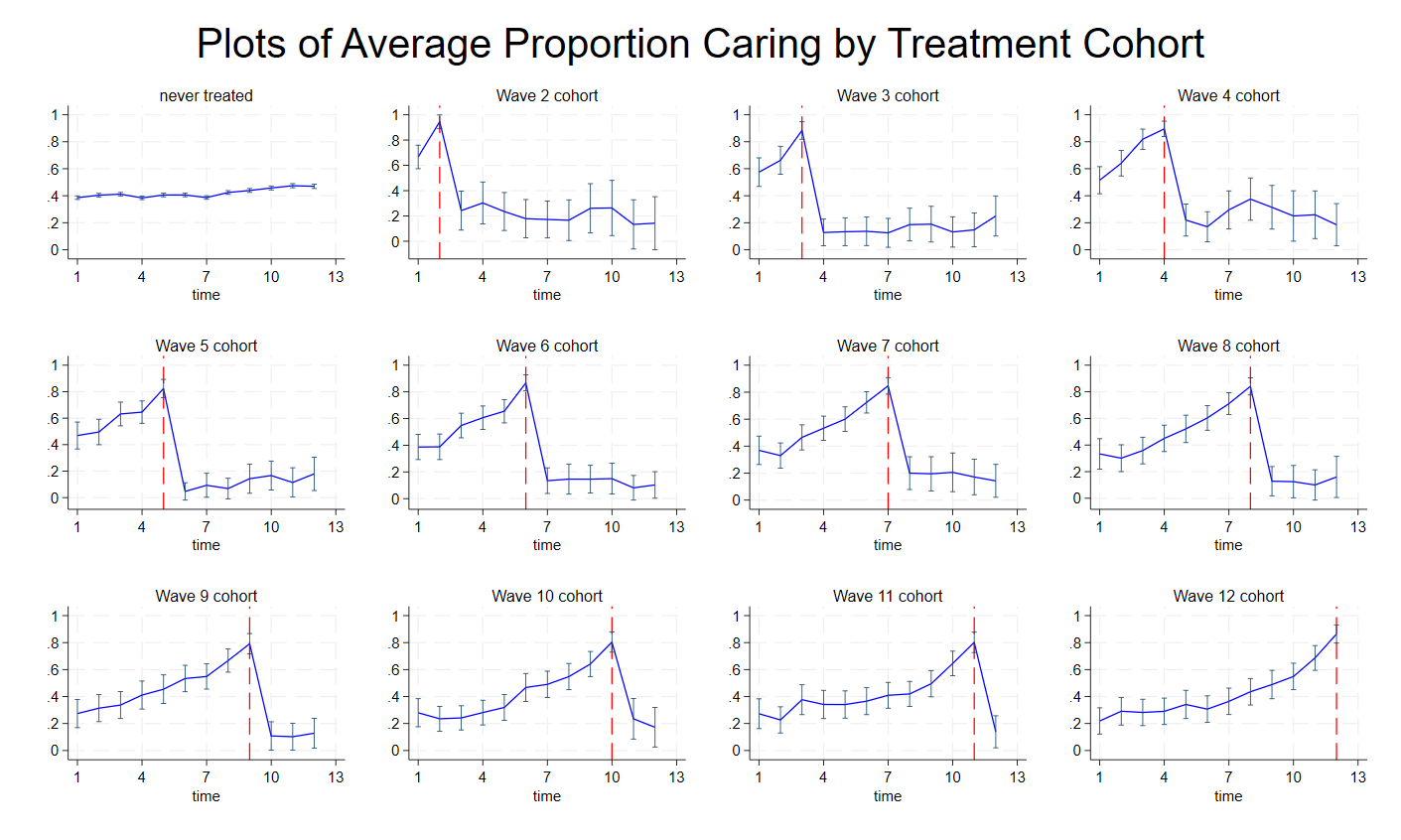


(Note: since carers can care for more than one person in the household, they can continue to be a carer after the deceased dies as they are caring for other household members).

Figure S7: Effect of bereavement on General Health Questionnaire (GHQ). Carers 1 = bereaved carers vs. non-bereaved carers. Carers 2 = bereaved carers vs. non-bereaved non-carers (household members who did not report providing care). Conditional event study.


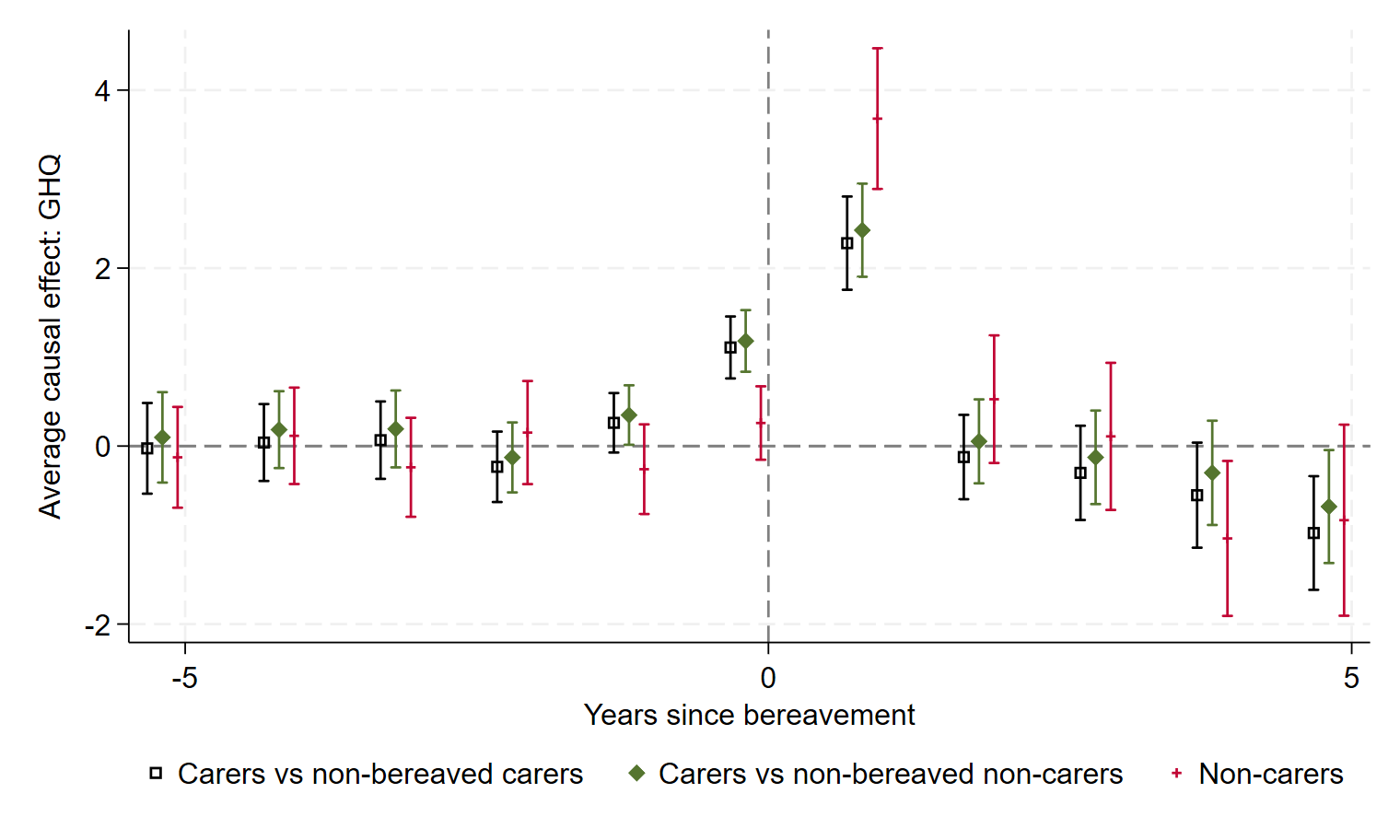


Figure S8: Effect of bereavement on SF-12 MCS. Carers 1 = bereaved carers vs. non-bereaved carers. Carers 2 = bereaved carers vs. non-bereaved non-carers (household members who did not report providing care). Conditional event study.


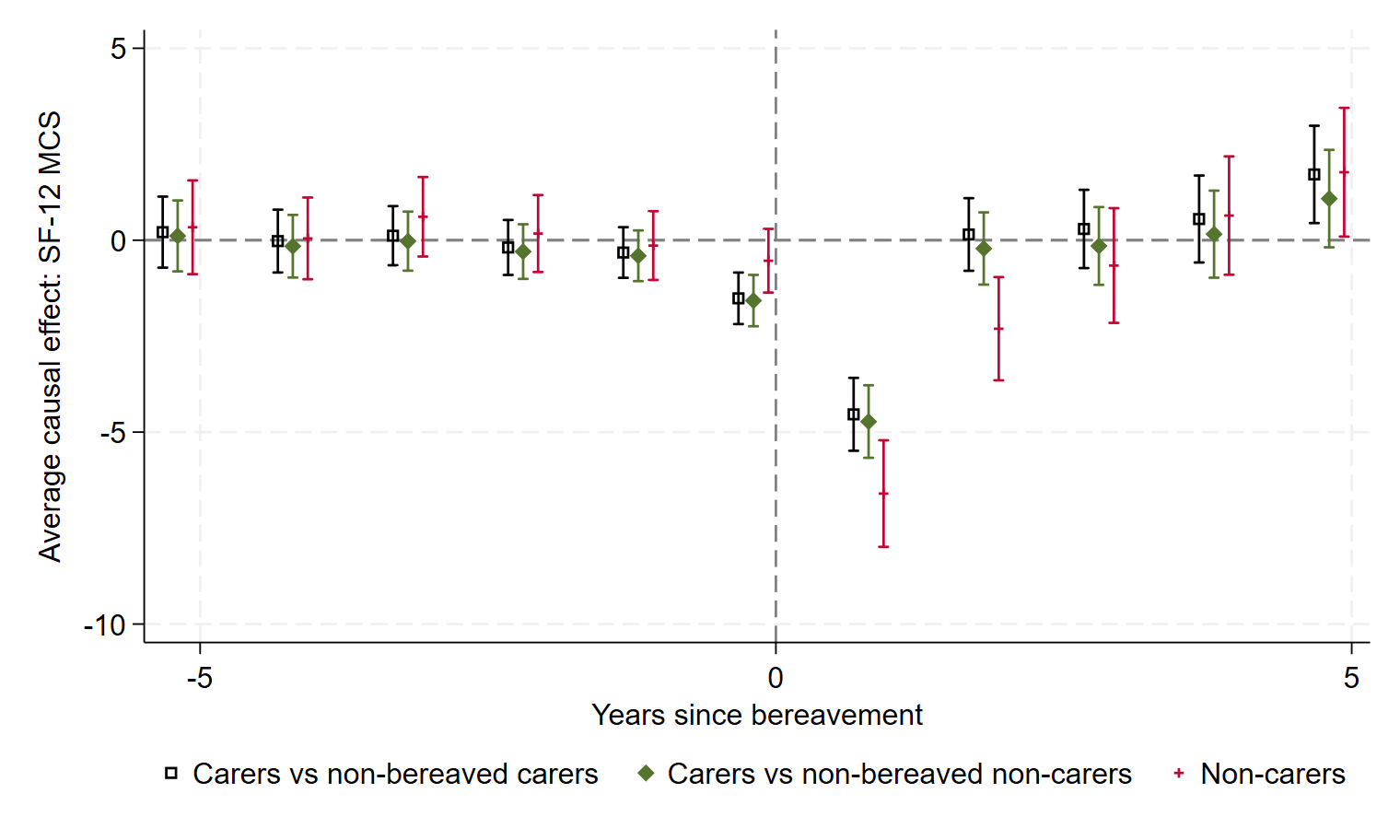


Figure S9: Effect of bereavement on SF-12 PCS. Carers 1 = bereaved carers vs. non-bereaved carers. Carers 2 = bereaved carers vs. non-bereaved non-carers (household members who did not report providing care). Conditional event study.


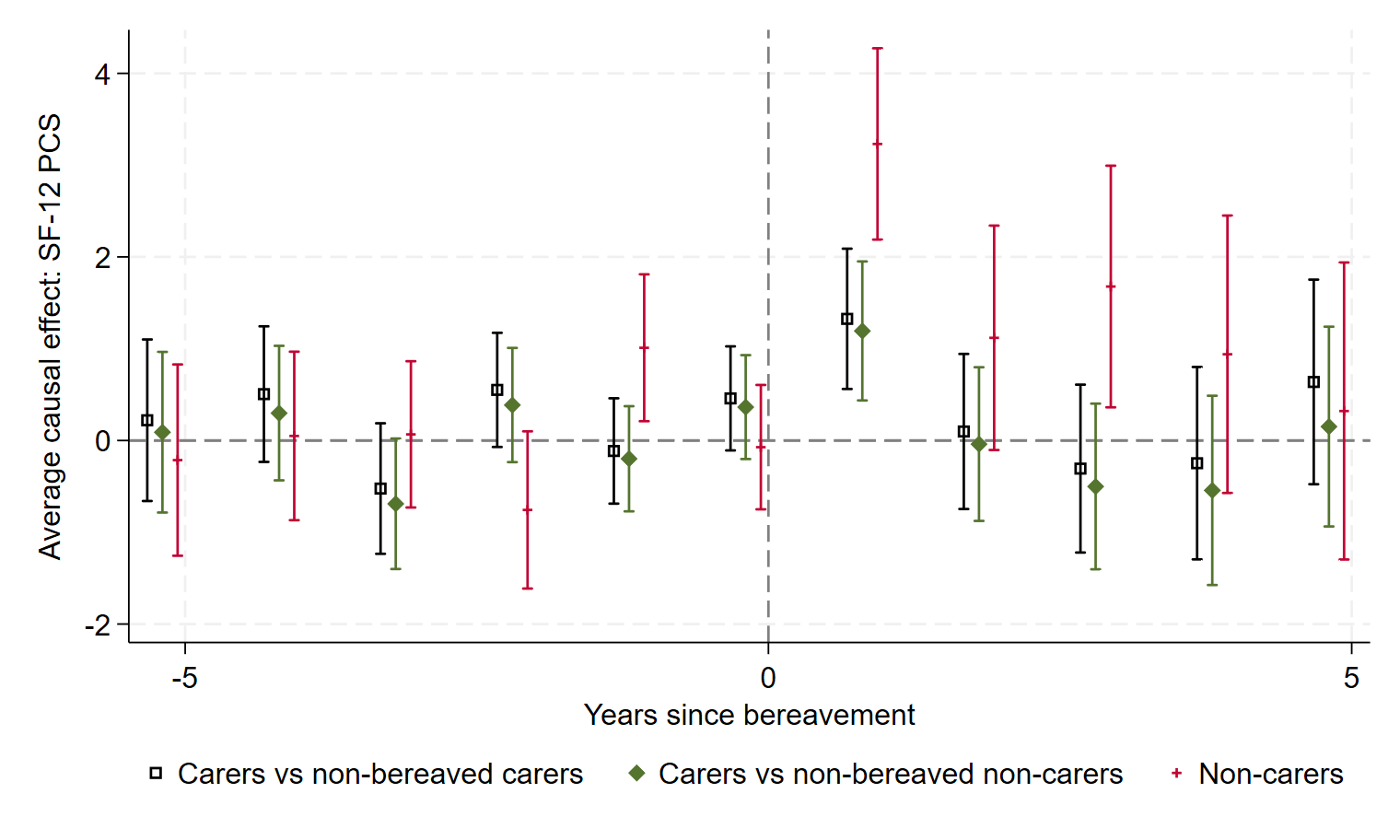


Figure S10: Responses to SF-6D domains for physical health, health limiting activities, pain, and energy, by time to bereavement for bereaved non-carers and bereaved carers.


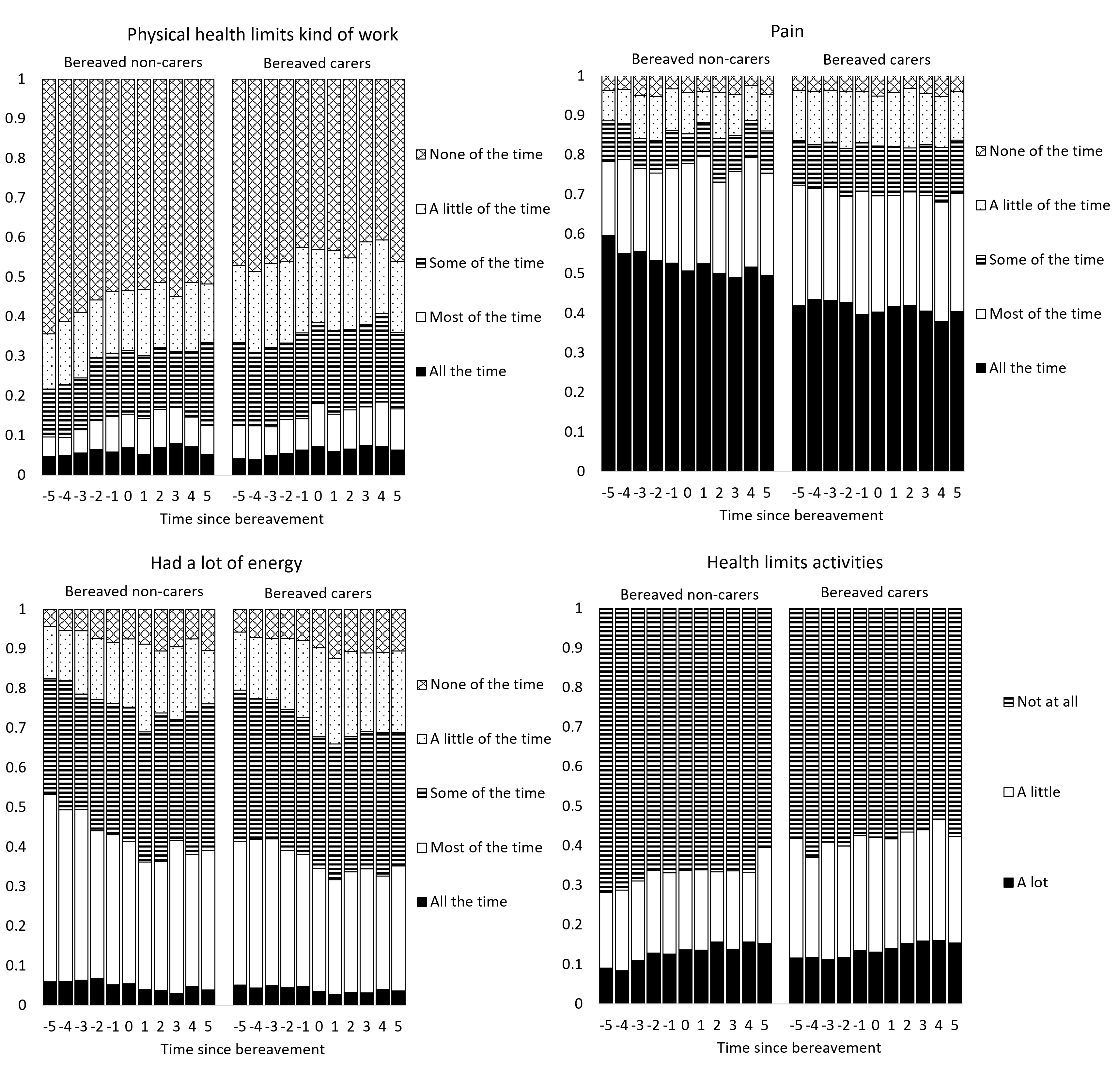


Figure S11: Effect of bereavement on SF-6D. In this analysis, only carers who cared for only one household member are included (carers who care for multiple household members are excluded). Carers 1b = bereaved carers vs. non-bereaved carers. Carers 2 = bereaved carers vs. non-bereaved non-carers (household members who did not report providing care).

Figure S12: Effect of bereavement on SF-6D. In this analysis, carers are only included if they provide care in the period before the event. Carers 1a = bereaved carers vs. non-bereaved carers. Carers 2 = bereaved carers vs. non-bereaved non-carers (household members who did not report providing care).
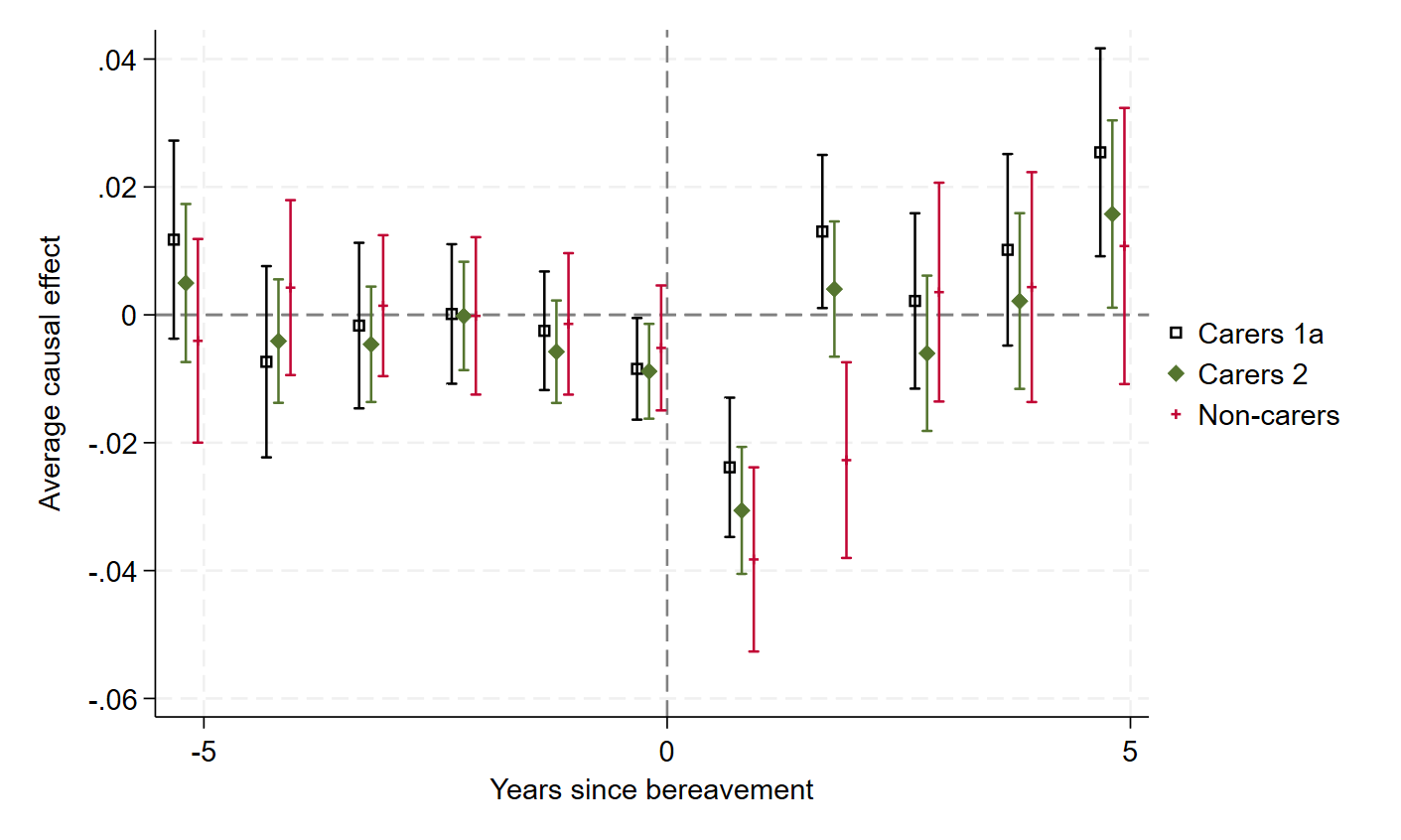


Figure S13: Effect of bereavement on General Health Questionnaire (GHQ), for subgroups based on the bereaved person’s relationship to the deceased.


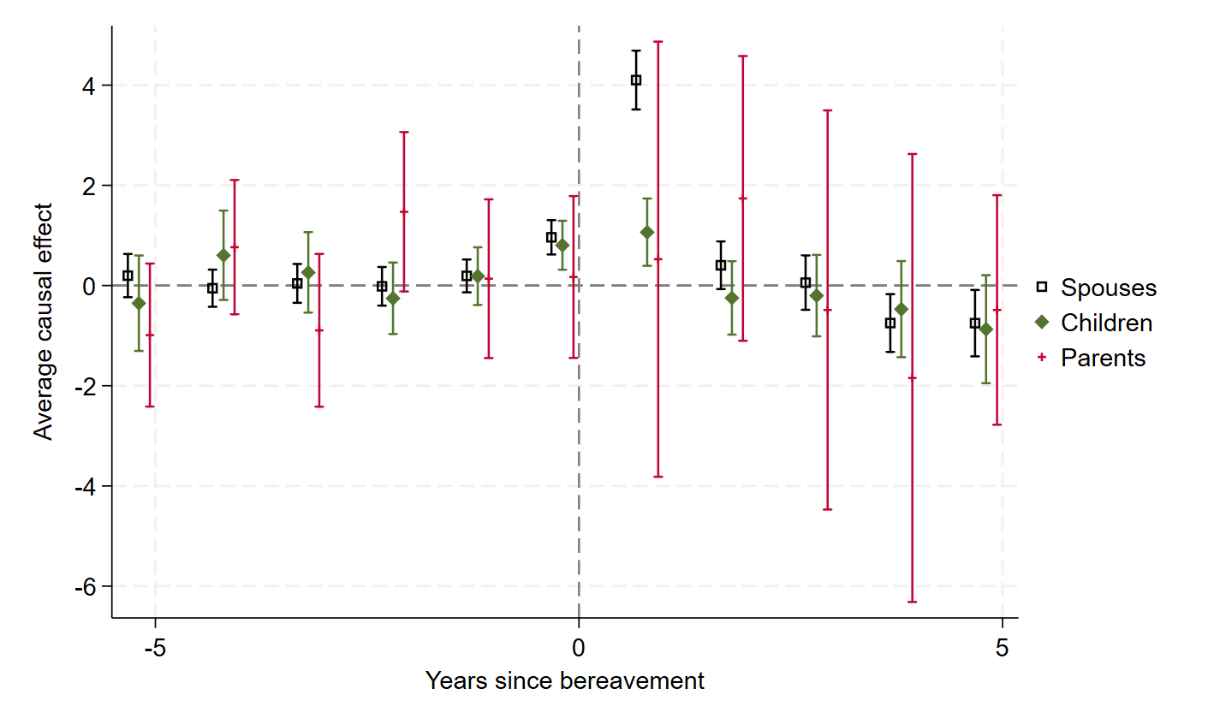


Figure 14: Distribution of deceased’s age at death by bereaved’s relation to deceased
